# Adapting the patient and physician versions of the 9-item shared decision making questionnaire for other healthcare providers in Japan

**DOI:** 10.1186/s12911-021-01683-8

**Published:** 2021-11-11

**Authors:** Yuko Goto, Yasuhiro Yamaguchi, Joji Onishi, Hidenori Arai, Martin Härter, Isabelle Scholl, Levente Kriston, Hisayuki Miura

**Affiliations:** 1 Department of Home Care and Regional Liaison Promotion, National Center for Geriatrics and Gerontology, 7-430 Morioka, Obu, Aichi 474-8511 Japan; 2 Department of Respiratory Medicine, Jichi Medical University Saitama Medical Center, Saitama, Japan; 3 Department of Community Healthcare and Geriatrics, Nagoya University Graduate School of Medicine, Nagoya, Japan; 4 National Center for Geriatrics and Gerontology, Obu, Japan; 5 Department of Medical Psychology, University Medical Center Hamburg-Eppendorf, Hamburg, Germany

**Keywords:** Patient-centered care, Shared decision-making, Healthcare provider, Interprofessional education, Reliability, Validity, Confirmatory factor analysis

## Abstract

**Background:**

In Japan, the number of older people with various health problems and difficulties in living is increasing. In order to practice patient-centered care for them, not only medical professionals but also multidisciplinary teams including care professionals and patients need to practice shared decision making (SDM) in the context of long-term care. For this reason, a measure of SDM in consultations with healthcare professionals (HCPs) other than physicians is needed. Therefore, this study aimed at adapting the patient and physician versions of the 9-item Shared Decision Making Questionnaire (SDM-Q-9, SDM-Q-Doc) for consultations with HCPs other than physicians in Japan.

**Methods:**

A pair of SDM measures that can be used by HCPs other than physicians, “Care SDM-Questionnaire for care receivers (SDM-C-patient)” and “Care SDM-Questionnaire for care providers (SDM-C-provider)” were prepared based on the Japanese versions of the SDM-Q-9 and SDM-Q-Doc. The internal consistency and conceptual structure of these measures were tested by secondary analysis of data from 496 participants from a workshop on SDM for different HCPs. Measurement invariance were tested by multigroup confirmatory factor analysis (CFA) for the patient (SDM-C-patient and SDM-Q-9) and provider (SDM-C-provider vs. SDM-Q-Doc) versions.

**Results:**

Both the Japanese SDM-C-patient and SDM-C-provider demonstrated high internal consistencies (Cronbach’s α coefficient was 0.90 and McDonald’s ω coefficient was 0.90 for both measures). CFA showed one-factor structures for both measures and original measures for physicians. Moreover, multigroup CFA showed configural and metric invariance between the novel care measures and original physician’s measures.

**Conclusions:**

Thus, the novel SDM measures for care providers in Japan as well as the original physician’s measures could be used in training setting. As these measures were tested only in a training setting, their reliability and validity as new measures for care should be tested in a clinical setting in future.

## Background

Due to increased uncertainty in medical care and diversity in patient values [1], the need for more shared decision making (SDM) has become apparent. This led to the incorporation of SDM into national health policies and guidelines [2, 3]. Therefore, SDM is known as the pinnacle of patient-centered care [4]. SDM is a communicative process where healthcare professionals (HCPs) and patients aim to reach decisions based on the best available evidence with a focus on supporting the patient to consider options and to achieve informed preferences [5]. In Japan, the society is aging faster than anywhere else in the world. The systems providing medical care and long-term nursing care need to be improved to meet the requirements of elderly patients who want to live a healthy life with their families, including the end-of-life period [6]. Patient-centered care requires improvement in decision making support for patients and the acquisition of skills for SDM by HCPs.

Although a team-based approach is recommended for SDM [7], currently, there are relatively many SDM studies related to treatment decisions by patients and physicians. According to a study on care-related decision support, SDM practice by a multidisciplinary team should include a care professional engaged in the area of palliative care for end-of-life patients, where the importance of supporting patients' quality of life is strongly recognized [8]. In addition, in palliative care studies for children and adolescents with cancer and their families, the importance of an approach involving decision-making support by multidisciplinary teams, including care professionals, has been pointed out [9]. In previous studies, SDM-Q-9 [10] and SDM-Q-Doc [11], which are SDM measures that have been translated and utilized in various languages around the world, were adapted for other groups of patients and professionals, including parents of sick children (PSDM-Q-PARENT) [12] and for nurses (PSDM-Q-NUR) [13]. The SDM measures have also been adapted to HCPs, but no indication for care professionals has been confirmed [14]. In Japan, the Medical Practitioners Act allows only doctors (physicians and dentists) to practice medical care, which includes prescribing medicines. The Act treats treatment decisions by physicians and care by HCPs other than physicians (e.g., assistance of medical practice, care support, welfare counseling, and rehabilitation) differently. Therefore, interprofessional healthcare teams, including physicians, work together to provide comprehensive patient care. In Japan, in recent years, the introduction of SDM by teams has been promoted in the recommendations for promoting ACP [15] and the guidelines for introducing hemodialysis therapy [16]. However, training on SDM skills is included in very few education programs offered to physicians and other HCPs in Japan [17]. An educational program related to SDM by Japanese nurses and patients has been developed, but SDM skill training for teams including care professionals has not been conducted [18]. The evaluation of these skill trainings for HCPs other than physicians is challenging because no SDM measures are currently available for this group in Japan. SDM measures for physicians and patients have been translated, adapted, and are already available in the Japanese context [19, 20]. However, these measures are difficult to use for other HCPs because of the unique characteristics of the Japanese language and the social systems, where the terms used in medical care and long-term care for the same object are often different.

This study aimed to evaluate the novel SDM measures designed for use in training settings with multiple healthcare providers in Japan, based on the existing SDM measures for physicians and patients.

## Methods

### Study design

The data obtained after conducting workshops to learn SDM support, held by a government agency in Aichi Prefecture, Japan, was used in this study. In the workshops, the novel SDM measures for HCPs other than physicians that we had developed in advance were used. The collected anonymized data was converted by a person in charge of information processing, who was not involved in the study, from the descriptive data of the “Care SDM-Questionnaire for care receivers (SDM-C-patient),” “Care SDM-Questionnaire for care providers (SDM-C-provider),” and “Japanese version SDM-Q-9/Japanese version SDM-Q-Doc” to electronic data. These data were used for analyses.

### SDM measures

The SDM-C-patient and SDM-C-provider were prepared based on the previously tested and published Japanese versions of the 9-item Shared Decision Making Questionnaire for patients (SDM-Q-9) [19] and physicians (SDM-Q-Doc) [20]. The original SDM-Q-9 (for patients) [10] and the SDM-Q-Doc (for physicians) [11] were developed in Germany (LK, MH, IS); the measures assess the subjectively experienced level of SDM from the patient’s and physician’s perspectives, respectively. The measures are based on a multicomponent model [21] constructed from the following four important elements of SDM [22]: (1) at least a patient and a professional participate; (2) information is shared by both parties [the patient and professional(s)]; (3) both parties [the patient and professional(s)] are aware of the availability and details of options; and (4) both parties [the patient and professional(s)] share decision making criteria and agree on the decision.

At the beginning of the questionnaire, there is a column to describe the purpose of the discussion and the content of the decision. The items are rated on a six-point Likert scale from “completely disagree” (0 points) to “completely agree” (5 points). The highest possible total score is 45 points. A higher score indicates a higher level of perceived SDM.

### Adaptation of the instrument in advance

**Fig. 1:**
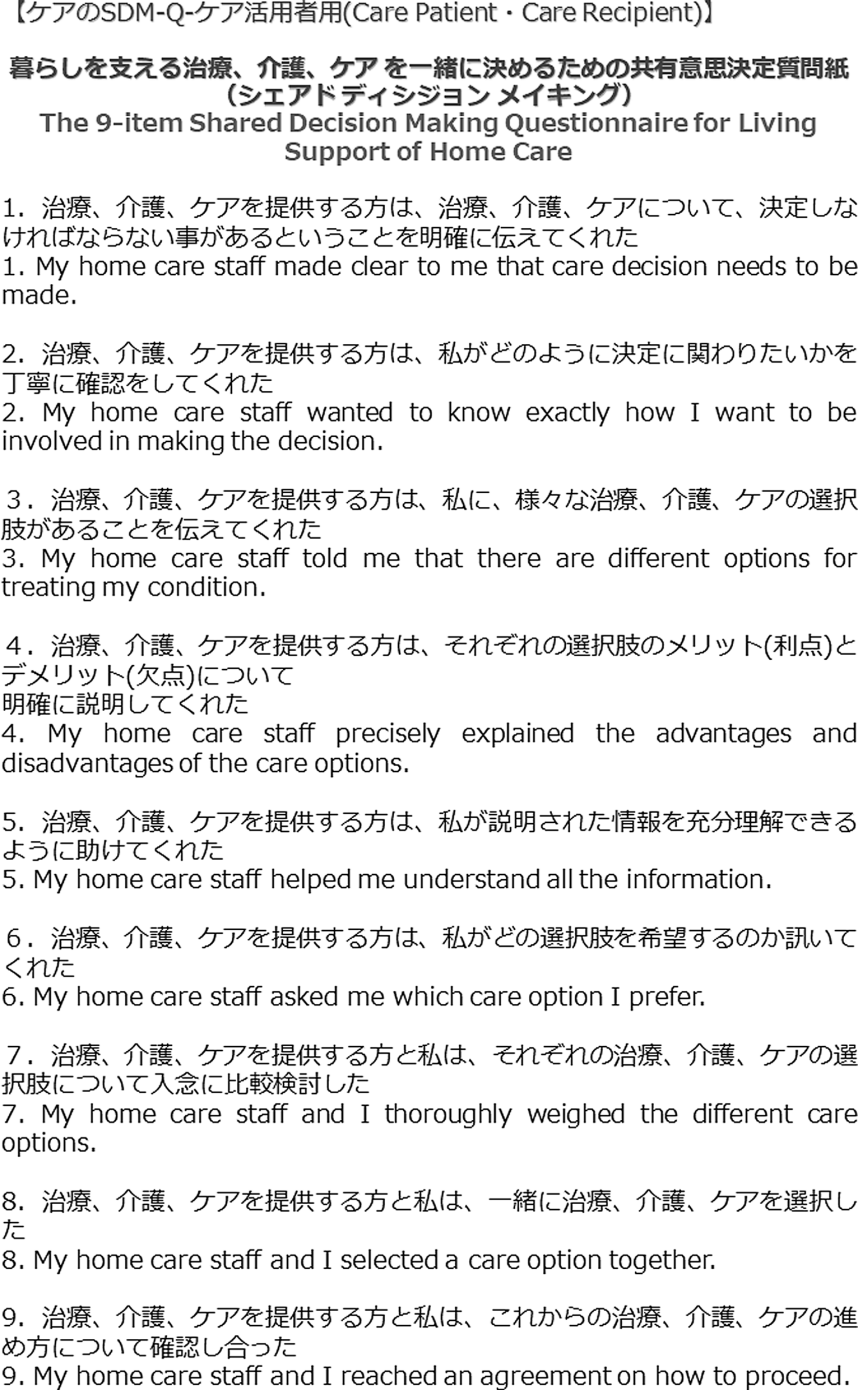
Items of the Japanese version of the SDM-C-patient and their reverse translations into English

After permission was obtained from the development team of the original SDM-Q-9 and SDM-Q-Doc, the team adapted the Japanese SDM-Q-9 and SDM-Q-Doc to care SDM measures using terms that were commonly understood by HCPs in Japan, considering the different terminologies between medical and long-term care, such as notations of “Kanja” (patient) or “Riyousha” (care receiver) in Japanese. The face validity of care SDM measures was tested with the help of the care provider managers in Japan. The Japanese authors created a tentative plan and revised it to ensure the validity and comprehension of the expression after receiving opinions from the nursing manager, therapist manager, and chief care manager of the Japanese native speaker who is also a researcher. Next, a researcher who has experience working as a care provider in an English-speaking country translated it from Japanese to English. Thereafter, the care SDM measures were translated to English and revised several times based on the advice received from the development team of the original SDM-Q-9 and SDM-Q-Doc (IS, LK, MH). Thus, the final Japanese versions of the SDM measures were approved by IS, LK, and MH (“SDM-C Japanese (Patient)” (Fig. 1) and “SDM-C Japanese (Care staff)” in reference [13]).

### Sample and setting

The data for secondary usage were collected from anonymized materials after the “Workshop to Learn Shared Decision-Making Support,” which was held by a government agency in Aichi Prefecture, Japan from September to November 2018. Medical (physicians, dentists, nurses, therapists, and pharmacists), long-term care (care managers and public healthcare nurses), and other (such as social workers) professionals attended the workshop as participants. The “care manager” plays a major role in care planning for care receiver under long-term care insurance in Japan. Therapists include physical, occupational, and speech therapists.

Workshop participants were recruited from local medical and long-term care professionals through recommendations of ten training sites (four hospitals, four municipalities, and two local medical associations) adopted by Aichi Prefecture. The workshop participants received a lecture on SDM and performed role-plays on cases presented in teams of three members each to learn SDM skills. In each team, one member played the role of a decision supporter, a different member played the role of a patient, and a third member played the role of an observer. We used several case vignettes such as decision support for people with cognitive impairment whose disease gradually progresses and activities of daily living gradually decline. As the case vignette, we used information from a single man with vascular dementia who lived alone and whose activities of daily living began to gradually decline. All role-playing teams worked on one patient model. Five decision-making vignettes had been prepared so that participants from various specialties could learn smoothly. Of these, One was a vignette in which a treatment decision had to be made at the time of outpatient examination, the second was a vignette in which the treatment decision had to be made in the hospital after a patient had a fall and had to be hospitalized on an emergency basis, the third was a vignette in which a decision regarding future care at home had to be made, the fourth was a vignette in which future medical treatment had to be made when a patient was entering a nursing home for a short period of time, and the fifth was a vignette in which a decision had to be made when a patient was receiving prescription drugs at a local family pharmacy. When the decision supporter was a physician, the Japanese SDM-Q-9 and SDM-Q-Doc were used to evaluate the treatment decision process. When the decision supporter was a HCP other than physicians, the SDM-C-patient and SDM-C-provider were used to evaluate the care decision process.

In these cases, professionals other than physicians acted as providers according to their profession, and other participants acted as patients and observers. After the role-play, the participants completed an appropriate SDM measure to evaluate the SDM process and discussed the possible improvements in patient–provider communications. After the workshop, the anonymized materials (role-play data using the SDM measures) were collected from ten training sites after the permission by the Aichi Prefecture, and the collected information was converted to electronic data by a person who was a staff member of the National Center for Geriatrics and Gerontology.

The descriptive data at the beginning of the SDM measures was converted into electronic data as it was. The Japanese versions of the SDM measures are six-point Likert-type scale questionnaires. Item scores were analyzed as “completely disagree,” which was scored as 0, and “completely agree,” which was scored as 5 points. We followed the development procedure of the original version and transformed the sum scale to range from 0 to 100 points. In this study, we used role-play data using SDM measures for those who played the patient and decision supporter roles for analysis.

The close fit model of covariance structure analysis was used to calculate the required sample size for the present study, based on the Japanese version of the SDM-Q-9 and SDM-Q-Doc [23]. The null hypothesis was the root mean square error of approximation (RMSEA) = 0, and the alternative hypothesis was RMSEA = 0.1. Based on the previous report on developing the Japanese version of SDM-Q-Doc [10], the degree of freedom, statistical power, and α error were set to 19, 0.8, and 0.05, respectively. Thus, the required sample size was 191 individuals.

### Statistical analyses

The highest possible score was 45 points for the Japanese versions of the SDM-C-patient, SDM-C-provider, SDM-Q-9, and SDM-Q-Doc; scores were converted to those in a full score of 100 points before descriptive statistical analysis.

Confirmative factor analysis (CFA) of the Japanese version of the SDM-C-patient and SDM-C-provider were conducted. CFA was conducted with the assumption that SDM-C-patient and SDM-C-provider have a one-factor structure because the Japanese version of SDM-Q-9 [19]/Japanese version of SDM-Q-Doc [20] had the same one-factor structure as the original SDM-Q-9/SDM-Q-Doc. The goodness of fit of the model was evaluated using chi-squared test, comparative fit index (CFI), RMSEA, goodness-of-fit index (GFI), adjusted GFI (AGFI), and Akaikeʼs information criterion (ACI). In the CFA, residual correlations were added one at a time in the descending order of correlation, and the addition was stopped when the CFI of ≥ 0.95, RMSEA of ≤ 0.05, and GFI/AGFI of ≥ 0.95 were attained.

To confirm the reliabilities of the Japanese versions of the SDM-C-patient and SDM-C-provider, Cronbach’s α coefficient and McDonald’s ω coefficient were calculated to confirm internal consistencies.

Measurement invariance was investigated by testing each of the two pairs of four different SDM measures among those who attended the same lecture at the same workshop. To evaluate the measurement invariance of the measures for patient (SDM-C-patient vs. SDM-Q-9) and provider (SDM-C-provider vs. SDM-Q-Doc), we used the multigroup CFA. Metric and scalar invariances were confirmed in the same manner as that used for the patient and provider versions. Chen’s criteria [24], where ∆CFI of ≤ 0.010 and ∆RMSEA of ≤ 0.015 indicate the presence of invariance, were used to judge invariance.

IBM SPSS Statistics 27, IBM SPSS Amos Graphics 27 (IBM Corp., Armonk, NY, USA), and R 4.0.2. (A language and environment for statistical computing.). R Foundation for Statistical Computing, Vienna, Austria. URL: https://www.R-project.org/) were used for analysis.

### Ethical considerations

This study involved the secondary usage of data provided after the workshops conducted at the training sites in Aichi Prefecture, and the analyzed data were entirely anonymized and included no information that could be used to identify specific individuals. Japan’s research ethics protocol, guidance on the ethical guidelines for medical and health research involving human subjects (revised March 23, 2021) [25], was published on April 16, 2021 [26]. To elaborate, in experiments and practical training conducted for academically known events such as health and hygiene training conducted exclusively for educational purposes, it is stated that if the obtained samples and data are not used for purposes other than educational ones, it may be judged that they do not fall under “research.”

This study is a secondary analysis of the information obtained from the training sessions conducted at the training sites in Aichi Prefecture, which is the main body responsible for conducting trainings.

At the time of the workshop, the following explanation was provided verbally and via slide presentation: “The training sites provide workshop information, which does not contain any personal identifiable information collected at the workshop, to another institution for training feedback and analysis.” For this study, the training sites collected information regarding the workshop and provided it to our researchers. Our researchers received the information that an individual could not be identified and the study was conducted using that information. In the third item, “scope of application,” of the ethical guideline for medical and health research involving human subjects (revised March 23, 2021) [25], information that has already been anonymized (limited to that by which a specific individual cannot be identified and a correspondence table has not been created) is excluded. Judgments outside the scope of this guideline were confirmed by several Japanese researchers who have received ethical education.

## Results

### Participant characteristics

Among no missing values of 779 participants, the data from 494 (247 pairs) who played the decision supporter or patient roles, except for the observer-role data, were included in the analysis (Table 1). Of those included, 404 (202 pairs) used SDM-Q for care receivers (SDM-C-patient) and SDM-Q for care providers (SDM-C-provider) in the care decision role-play, whereas 90 (45 pairs) used the SDM-Q-9 and SDM-Q-Doc in the treatment decision role-play. Of a total of 779 participants, 340 (45%) were nurses, 90 (12%) were medical social workers, 89 (11%) were care managers, 79 (10%) were physicians, 34 (4%) were pharmacists, 22 (3%) were therapists, and 125 (15%) were others (Table 1). The participants were divided into groups as per the years of clinical experience (by 5 years), and those with ≥ 25 years of experience accounted for 25%, thus indicating that participants with various lengths of experience attended the workshop (Table 1). Others included public health nurses, life counselors, long-term care workers, and certified care workers.

**Table 1:** Professions and years of clinical experience of the participants

	Number	%
*Professions of participants*		
Nurse	340	45
Medical social worker	90	12
Care manager	89	11
Physician	79	10
Pharmacist	34	4
Therapist	22	3
Others	125	15
Total	779	100
*Years of clinical experience*		
< 5	90	12
5–9	108	14
10–14	126	16
15–19	111	14
20–24	137	18
> 25	193	25
No answer	14	1
Total	779	100

### Care SDM-Q for care receivers (SDM-C-patient)

**Fig. 2:**
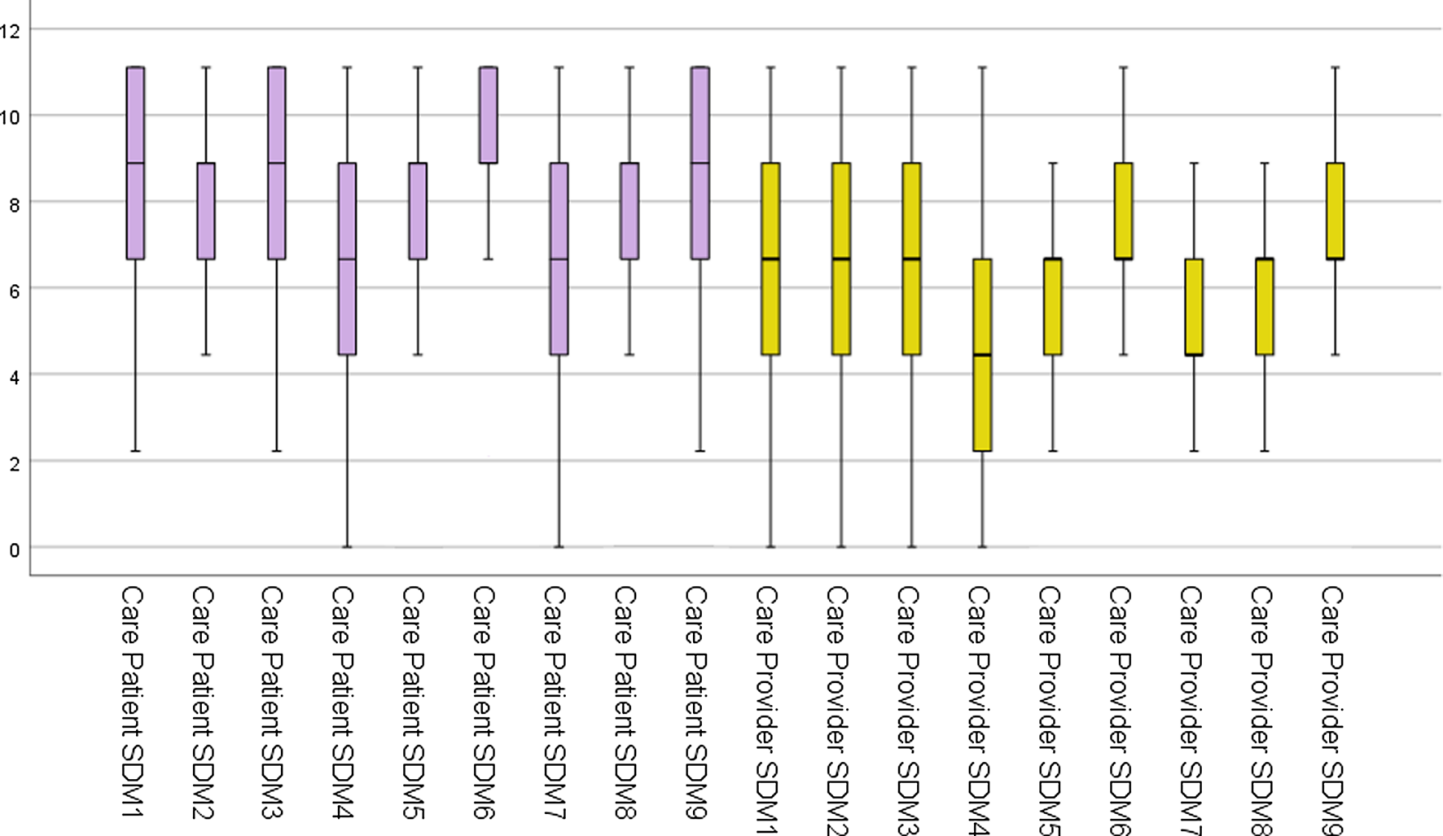
Values for items of the Japanese version SDM-C-patient and SDM-C-provider (Box and Whisker Plot)

The results of care SDM-Q for care receivers are shown in Table 2, Fig. 2.

**Table 2:** Descriptive statistics for SDM-C-patient (n = 202)

	Median	Mean	SD	Minimum	Max	Corrected item-total correlation coefficient
Item 1	8.89	8.35	2.26	2.22	11.11	0.47
Item 2	8.89	8.36	2.15	2.22	11.11	0.51
Item 3	8.89	8.50	2.24	2.22	11.11	0.45
Item 4	6.67	6.31	2.41	0.00	11.11	0.52
Item 5	8.89	8.36	2.16	0.00	11.11	0.57
Item 6	8.89	9.14	1.94	2.22	11.11	0.40
Item 7	6.67	6.85	2.43	0.00	11.11	0.56
Item 8	8.89	8.31	2.26	0.00	11.11	0.62
Item 9	8.89	8.34	2.23	0.00	11.11	0.55

**Fig. 3:**
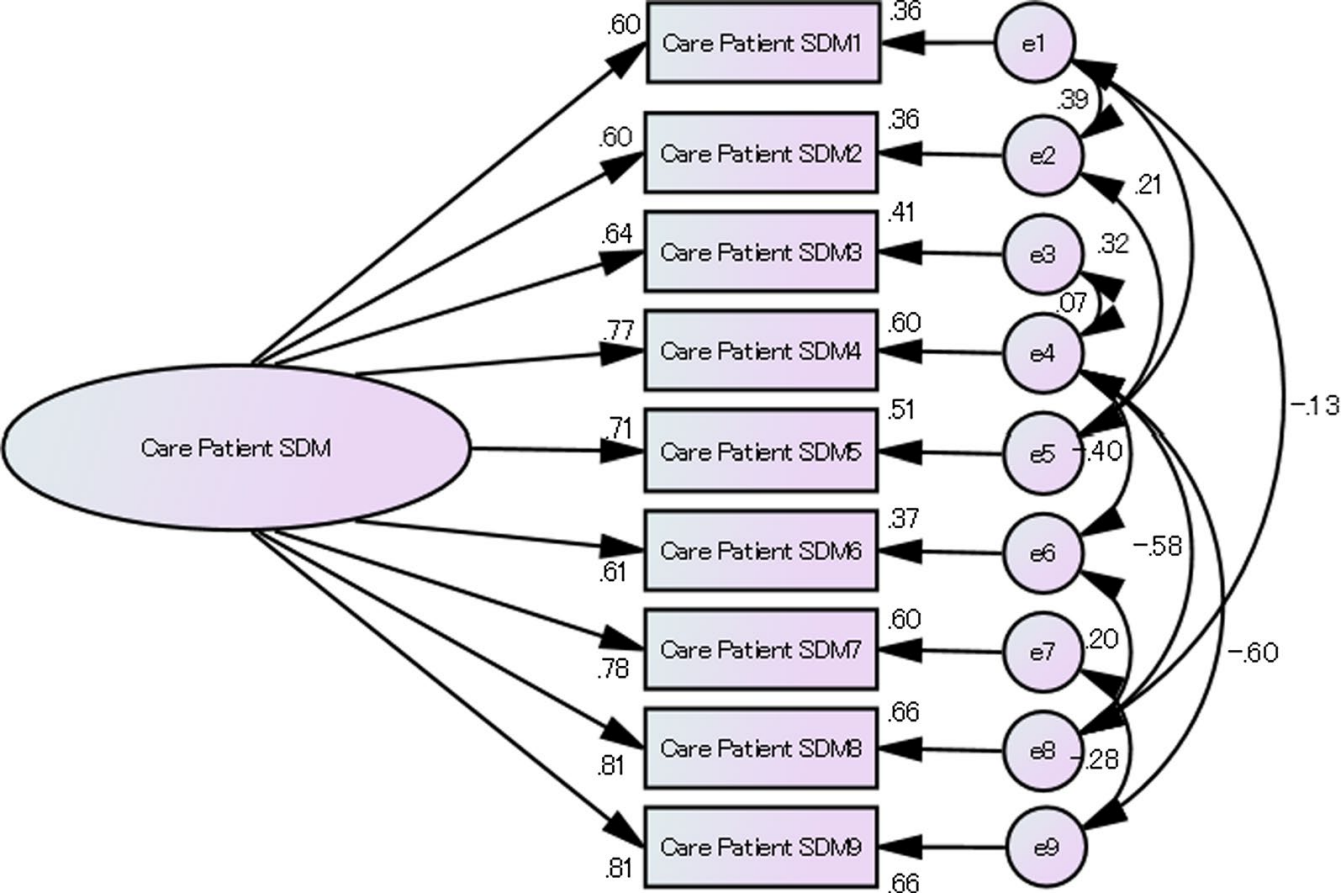
Factor structure of SDM-C-patient

For all nine items, the corrected item-total correlation coefficient was ≥ 0.40. In CFA, the fit of the one-factorial model with no residual correlations was poor. We then constructed a second model allowing residual correlation and confirmed that it had satisfactory goodness of fit (Table 3, Fig. 3).

**Table 3:** CFA for SDM-C-patient and provider models

	χ^2^ value (*p*)	DF	CFI	RMSEA	GFI	AGFI	ACI
*CFA for SDM-C-patient models*							
Model 1 (without residual correlation)	123.83 (0.00)	27	0.89	0.13	0.87	0.78	159.83
Model 2 (with residual correlations)	16.73 (0.47)	17	1.00	0.00	0.98	0.95	72.73
*CFA for SDM-C provider models*							
Model 3 (without residual correlation)	68.00 (0.00)	27	0.95	0.09	0.93	0.88	104.00
Model 4 (with residual correlations)	21.75 (0.59)	24	1.00	0.00	0.98	0.96	63.75

CFA, confirmatory factor analysis; SDM-C-patient, care SDM-Questionnaire for care receivers; SDM-C-provider, care SDM-Questionnaire for care providers; CFI, comparative fit index; RMSEA, root mean square error of approximation; GFI, goodness-of-fit index; AGFI, adjusted GFI; ACI, Akaikeʼs information criterion; DF, degree of freedom.

### Care SDM-Q for care providers (SDM-C-provider)

The results of SDM-C-provider are shown in Table 4, Fig. 2. The corrected item-total correlation coefficient for all nine items of care provider was ≥ 0.40 as was the case for care patient.

**Table 4:** Role for care provider’s descriptive statistics (n = 202)

	Median	Mean	SD	Minimum	Maximum	Corrected item-total correlation coefficient
Item 1	6.67	6.59	2.62	2.22	0.00	0.44
Item 2	6.67	6.27	2.34	2.22	0.00	0.42
Item 3	6.67	6.48	2.29	2.22	0.00	0.52
Item 4	4.44	4.62	2.32	0.00	0.00	0.40
Item 5	6.67	6.34	2.08	0.00	0.00	0.49
Item 6	6.67	6.91	2.28	2.22	0.00	0.54
Item 7	4.44	5.13	2.13	0.00	0.00	0.52
Item 8	6.67	6.19	2.04	0.00	0.00	0.56
Item 9	6.67	6.77	2.34	0.00	0.00	0.51

**Fig. 4:**
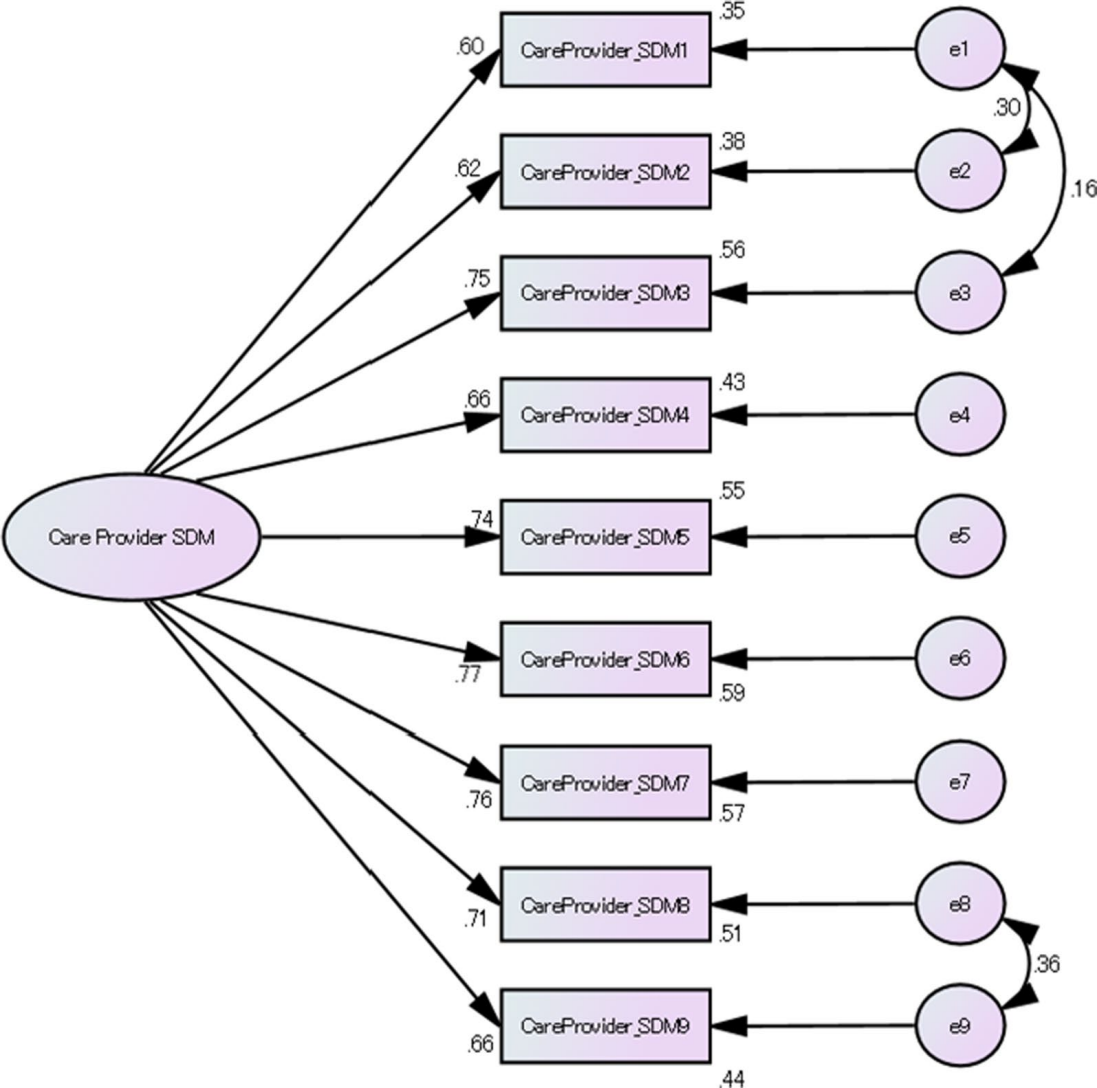
Factor structure of SDM-C-provider

The fit of the model without residual correlations was poor. We then constructed a model allowing residual correlations and confirmed satisfactory goodness of fit (Table 3, Fig. 4).

### Reliability analysis of care SDM

For care SDM-Q for care receivers (SDM-C-patient), Cronbach’s α coefficient was 0.90 and McDonald’s ω coefficient was 0.90.

For care SDM-Q for care providers (SDM-C-provider), Cronbach’s α coefficient was 0.90 and McDonald’s ω coefficient was 0.90.

#### Measurement invariance of the SDM-C-patient and SDM-Q-9

The measurement invariance of SDM-C-patient was used as an evaluation scale by 202 participants who played the care patient role and the Japanese version of SDM-Q-9 was used by 45 participants who played the patient role. The results are presented in Table 5.

**Table 5:** Analysis of measurement invariance of the care versions and the original physician’s versions of measures

Model	χ^2^(Δχ^2^)	df(Δdf)	*p*(*p*-value of the difference)	CFI (ΔCFI)	RMSEA (ΔRMSEA)
*Patient role group (SDM-C-patient vs SDM-Q-9)*					
Configural	170.287	54	< 0.001	0.886	0.094
Metric	(11.153)	(8)	(0.193)	(0.003)	(-0.005)
Scalar	(32.371)	(17)	(0.014)	(0.120)	(-0.002)
*Provider role group (SDM-C-provider vs SDM-Q-Doc)*					
Configural	183.137	54	< 0.001	0.878	0.099
Metric	(16.441)	(8)	(0.036)	(0.008)	(-0.004)
Scalar	(40.337)	(17)	(0.001)	(0.140)	(-0.001)

CFI, comparative fit index; RMSEA, root mean square error of approximation; SDM-C-patient, care SDM-Questionnaire for care receivers; SDM-Q-9, 9-item Shared Decision Making Questionnaire for patients; SDM-C-provider, care SDM-Questionnaire for care providers; SDM-Q-Doc, 9-item Shared Decision Making Questionnaire for physicians

Multigroup CFA showed no significant difference between factor loadings of nine corresponding items of the SDM-C-patient and SDM-Q-9. The results of valuing configural invariance indicated a good fit to data [χ^2^(54) = 170.287, *p* < 0.001; CFI = 0.886; RMSEA = 0.094]. Thus, configural invariance was demonstrated.

Thereafter, metric invariance was constructed by first assuming that the two groups had the same factor loadings. Scalar invariance was then constructed assuming that the two groups had the same item intercepts. In testing metric invariance, ΔCFI was 0.003 (< 0.010) and ΔRMSEA was − 0.005 (< 0.015), but in testing scalar invariance, ΔCFI was 0.120 (> 0.010).

These results showed that metric invariance was demonstrated but scalar invariance was not.

### Measurement invariance of the SDM-C-provider and SDM-Q-Doc

The measurement invariance of SDM-C-provider was used by 202 participants who played the care provider role and the Japanese version of SDM-Q-Doc was used by 45 participants who played the physician role. The results are presented in Table 5. Multigroup CFA showed no significant difference between factor loadings of nine corresponding items of the SDM-C-provider and SDM-Q-Doc. The results of valuing configural invariance indicated a good fit to data [χ^2^(54) = 183.137, *p* < 0.001; CFI = 0.878; RMSEA = 0.099]. Thus, configural invariance was demonstrated.

In testing metric invariance, ΔCFI was 0.008 (< 0.010) and ΔRMSEA was − 0.004 (< 0.015), but in testing scalar invariance, ΔCFI was 0.140 (> 0.010). These results showed metric invariance was demonstrated but scalar invariance was not.

## Discussion

To promote SDM in long-term care setting in Japan, the novel assessment measure to evaluate care professionals SDM skill is needed, whereas the assessment measure in medical care setting in Japan is already developed. Therefore, we adapted the following two existing Japanese measures for physicians to measure SDM in consultations with HCPs other than physicians: SDM-C-patient and SDM-C-provider.

### Discussion on the measures for HCPs and its comparison with original measures

The results demonstrated that the pair of care SDM measures (for care receivers and care providers) developed in advance had a one-factor structure as the SDM measures for patient/physician did and both showed high internal consistencies. CFA demonstrated that the goodness of fit was poor unless the model assumed multiple residual correlations, which is a result similar to that for the patient/physician versions of SDM measures [27]. Acceptable levels of goodness of fit were obtained for the original SDM-Q-9/SDM-Q-Doc developed in Germany when multiple residual correlations were assumed. The requirement for this assumption has been suggested to be attributable to substantial construct heterogeneity [23].

Multigroup CFA showed configural and metric invariance between the novel and original measures (SDM-C-patient vs SDM-Q-9 and SDM-C-provider vs SDM-Q-Doc) according to Chen’s criteria [24]. It is thus possible to compare the mean scores of the factors between the novel care version and the original patient/physician version.

In order to support patients’ decision-making, it is necessary to form a team of multidisciplinary professionals, and the team members need to have similar levels of decision-making skills. However, there remains a difference between the terms used in case of SDM for the purpose of facilitating treatment decisions by patients and physicians and those used in case of SDM for the purpose of facilitating care decisions by patients and care professionals. Therefore, in Japan, it has not been possible to measure and evaluate both with the same measure.

Using the statistical method of measurement invariance testing, which is widely used in the field of psychology, to verify the homogeneity of SDM measurements obtained from medical and care professionals having different cultural backgrounds is very important to promote a decision making by a multidisciplinary team. The verification of invariance ensures that the team’s evaluation is homogeneous [28].

Recommended practices have been presented for conducting tests of measurement invariance [29]. In this study, first, we performed CFA via structural equation modeling and confirmed the configural invariance by evaluating the equivalence of factor structures in which the number of factors and the observed variables of each factor were the same. Next, the measurement invariance was verified. Measurement invariance is assessed in stages. Metric invariance is a state in which the loading of each observing factor is equal between the two groups, and scalar invariance is a state in which the intercept of each observing variable is equal in addition to metric invariance. The evaluation of the measurement invariance model is still under study [30]. It has been pointed out that changes in the χ^2^ value are easily affected by the sample size and sample characteristics [31], and in this study we evaluated the changes of the alternative fit indexes, CFI and RMSEA, in addition to the χ^2^ value [24, 32]. Since homogeneity can change depending on the translation and the usage of words in each population, it was not found to be a very high-fit model in this study; therefore, some cross-cultural differences between the two populations may exist. As a result, further research is required to clarify the differences in term recognition between the two populations. In this study, it was essential to confirm the equivalence of factor structures in which the number of factors and the observed variables of each factor are the same among medical and care professionals.

### Discussion on the measures in the SDM training setting

In other countries, attempts have been made to use these scales for actual SDM training [33–35]. As older patients are increasing and the number of patients requiring interventions for mental and social problems is rapidly increasing, SDM training tools that can be commonly used by professionals in different job categories are required to support patient decision-based long-term care by interprofessional care teams including physicians. The configural and metric invariance of SDM measures for care providers and physicians suggested in this study raises the hopes of promotion of training on SDM by interprofessional care teams including physicians.

Although there is a problem that SDM skill training for multidisciplinary teams is not actively progressing [36], the developed instruments can be used to support and evaluate SDM training for HCPs other than physicians. We anticipate that these measures can be used in clinical practice as well, which needs further testing in clinical settings.

In Japan, the construction of a community-based integrated care system is being developed as a policy, and it is beneficial to promote it for developing multidisciplinary teams on a regional basis. This time, by witnessing the transformation of participants through SDM skill training using measures having verified homogeneities, it will be possible to enable the construction of an effective educational program. A more appropriate educational program can be developed via the evaluation of educational programs, including SDM skill training for multidisciplinary teams, and by rigorously advancing training evaluation using the New World Kirkpatrick Model, which is widely used in medical and nursing education [37–40].

In Japan, it is necessary to promote education among multidisciplinary teams, and at the same time, actively promote SDM training for care professionals who belong to a large population and where SDM is not widely used. Differences between the SDM skills of the two populations, i.e., medical and care professionals, can affect the changes in SDM skills. Therefore, in order to support effective SDM practice by multidisciplinary teams, it is necessary to review the management and educational programs to compare the differences between the two populations in terms of SDM skills and bridge the differences. Implementing such efforts will lead to a steady acceleration of SDM practice by multidisciplinary teams even in Japan where the development of SDM practice is lagging.

## Limitations

Larger sample size is required to better power for the analysis of CFA and multigroup CFA. However, we did not obtain additional sample because this study involved the secondary usage of data after conducting the workshops.

In this study, reliability and structural validity were confirmed based on the data from the workshop in which only professionals with clinical experience participated. Therefore, the results apply presumably to HCPs who saw the requirement to learn SDM, were interested in SDM, and were able to understand the technical terms. These characteristics of the participants may have affected SDM role-play. In the future, investigations in actual clinical settings with scales to measure the concurrent construct validity of SDM should be conducted to confirm the reliability, criterion-related validity, and convergent validity again.

## Conclusion

In a setting for training HCPs, reliability and validity of the novel SDM measures developed for care providers were supported. In the future, it will be necessary to carry out further tests related to decision-making support of patients and care professionals. As these measures were tested only in a training setting, their reliability and validity as new measures for care should be tested in a clinical setting in the future.

## Abbreviations

ACI: Akaikeʼs information criterion
AGFI: Adjusted goodness-of-fit index
CFA: Confirmatory factor analysis
CFI: Comparative fit index
HCP: Healthcare professional
GFI: Goodness-of-fit index
RMSEA: Root mean square error of approximation
SDM: Shared decision-making
SDM-C-patient: Care SDM-Questionnaire for care receivers
SDM-C-provider: Care SDM-Questionnaire for care providers
SDM-Q-9: 9-Item Shared Decision-Making Questionnaire for patients
SDM-Q-Doc: 9-Item Shared Decision-Making Questionnaire for physicians

## Declarations

### Availability of data and materials

The data supporting the findings of this study are available from the corresponding author YG on request.
